# Bardoxolone-Methyl (CDDO-Me) Suppresses Androgen Receptor and Its Splice-Variant AR-V7 and Enhances Efficacy of Enzalutamide in Prostate Cancer Cells

**DOI:** 10.3390/antiox9010068

**Published:** 2020-01-12

**Authors:** Namrata Khurana, Partha K. Chandra, Hogyoung Kim, Asim B. Abdel-Mageed, Debasis Mondal, Suresh C. Sikka

**Affiliations:** 1 Department of Urology, Tulane University School of Medicine, 1430 Tulane Avenue, New Orleans, LA 70112, USA; nkhurana@wustl.edu (N.K.); hkim8@tulane.edu (H.K.); amageed@tulane.edu (A.B.A.-M.); 2 Department of Pharmacology, Tulane University School of Medicine, 1430 Tulane Avenue, New Orleans, LA 70112, USA; pchandr1@tulane.edu; 3 Department of Internal Medicine-Medical Oncology, Washington University in St. Louis Medical Campus, 660 S Euclid Ave, St. Louis, MO 63110-1010, USA; 4 Department of Microbiology, Lincoln Memorial University—Debusk College of Osteopathic Medicine, 9737 Coghill Drive, Knoxville, TN 37932, USA

**Keywords:** bardoxolone methyl, prostate cancer, castration-resistant prostate cancer, androgen receptor (AR), AR-V7, anti-androgen, enzalutamide, androgen deprivation therapy

## Abstract

Androgen receptor (AR) signaling is fundamental to prostate cancer (PC) progression, and hence, androgen deprivation therapy (ADT) remains a mainstay of treatment. However, augmented AR signaling via both full length AR (AR-FL) and constitutively active AR splice variants, especially AR-V7, is associated with the recurrence of castration resistant prostate cancer (CRPC). Oxidative stress also plays a crucial role in anti-androgen resistance and CRPC outgrowth. We examined whether a triterpenoid antioxidant drug, Bardoxolone-methyl, known as CDDO-Me or RTA 402, can decrease AR-FL and AR-V7 expression in PC cells. Nanomolar (nM) concentrations of CDDO-Me rapidly downregulated AR-FL in LNCaP and C4-2B cells, and both AR-FL and AR-V7 in CWR22Rv1 (22Rv1) cells. The AR-suppressive effect of CDDO-Me was evident at both the mRNA and protein levels. Mechanistically, acute exposure (2 h) to CDDO-Me increased and long-term exposure (24 h) decreased reactive oxygen species (ROS) levels in cells. This was concomitant with an increase in the anti-oxidant transcription factor, Nrf2. The anti-oxidant N-acetyl cysteine (NAC) could overcome this AR-suppressive effect of CDDO-Me. Co-exposure of PC cells to CDDO-Me enhanced the efficacy of a clinically approved anti-androgen, enzalutamide (ENZ), as evident by decreased cell-viability along with migration and colony forming ability of PC cells. Thus, CDDO-Me which is in several late-stage clinical trials, may be used as an adjunct to ADT in PC patients.

## 1. Introduction

Prostate cancer (PC) is the second leading cause of cancer-related mortality in men in the United States [1]. Notwithstanding the initial efficacy of androgen deprivation therapy (ADT), outgrowth of castration-resistant prostate cancer (CRPC) is the primary cause of death among patients [2]. The development of CRPC is linked with continuous androgen receptor (AR) signaling even in the absence of androgens [3,4,5,6,7]. Several mechanisms responsible for the constitutive AR signaling in CRPC cells include AR gene amplification, ligand-independent AR activation by cytokines or kinases, both intracrine and/or intratumoral androgen production, overexpression of AR co-activators, and most importantly, the expression of constitutively active AR splice variants (AR-Vs) [8,9]. Despite the castrated levels of androgens, these spliced forms of AR lacking the C-terminal ligand binding domain (LBD), promote the transcriptional activation of AR target genes as they still retain the transactivating N-terminal domain (NTD) [8,9,10]. 

AR-V7 (also known as AR3) is the most significant functional protein encoding AR splice variant [11,12,13,14,15,16,17,18,19]. Augmented levels of AR-V7 were identified in CRPC tumor specimens [18] and circulating tumor cells [13]. Elevated AR-V7 expression was found after the development of CRPC tumors when primary tumor tissues were examined before and after the development of castration resistance [11,14,15,16,17,18,19]. Moreover, overexpression of AR-V7 is one of the key factors in the development of resistance to the potent second-generation anti-androgens, e.g., enzalutamide (ENZ) and abiraterone acetate (ABI) [20,21]. Studies have also shown a critical role of full-length AR (AR-FL) in dimerizing and transactivating AR-V7 [22], which is involved in castration-resistant cell growth [23]. Therefore, there is a critical requisite for potential therapeutic strategies which can efficiently reduce AR-FL and AR-V7 linked constitutive tumor promoting signaling in the CRPC cells. 

Bardoxolone-methyl, the C-28 methyl ester of 2-cyano-3,12-dioxoolean-1,9-dien-28-oic acid (CDDO) known as CDDO-Me or RTA 402 is one of the synthetic triterpenoids that has been shown to have anti-inflammatory, as well as anticarcinogenic activities [24,25]. Studies with CDDO-Me have been conducted in various kinds of cancers such as prostate [26], breast [27], ovary [28], lung [29], leukemia [30], pancreatic [31], and osteosarcoma [32]. CDDO-Me activates Keap1/Nrf2/ARE pathway [33,34], inhibits nuclear factor kappa-B (NF-ĸB) [35] and Janus-activated kinase (JAK)/STAT (signal transducer and activator of transcription) pathway [36], and is effective at low nanomolar concentrations [24]. The α, β-unsaturated carbonyl groups present on its rings form reversible adducts with the thiol groups of critical cysteine residues in target proteins such as Keap-1 and inhibitor of kappa-B (IĸB) kinase (IKKβ). The binding of CDDO-Me to Keap1 releases Nrf2 impeding its ubiquitination, thus leading to the stabilization and nuclear import of this potent transcription factor [24]. Activated Nrf2 reduces intracellular reactive oxygen species (ROS) levels via the transcriptional induction of numerous antioxidant proteins, e.g., superoxide dismutase (SOD) and glutathione peroxidase (GPX) leading to a synchronized antioxidant and anti-inflammatory response [33]. Similarly, when CDDO-Me binds to IKKβ, it prevents NF-ĸB dissociation from its bound complex with IĸB in the cytosol, thus resulting in the suppression of NF-ĸB activation and the downstream cascade of pro-inflammatory signaling pathways [35]. Various other mechanisms responsible for the anticancer action of CDDO-Me involve inhibition of proliferation of cancer cells, induction of apoptosis, and arrest of cancer cells in the G_2_/M phase [30,37,38,39]. In vivo studies have also reported potent inhibitory effects of CDDO-Me on tumor growth, metastasis and angiogenesis [40,41]. CDDO-Me has demonstrated promising anticancer effects in Phase I clinical trials against multiple solid tumors [42]. Although multiple studies with CDDO-Me have been conducted in PC [26,37,41,43,44], its efficacy to suppress the expression of both AR-FL and AR-V7 in PC cells has not been investigated before.

In the current study, we have shown that at physiologically achievable plasma concentrations (i.e., nanomolar doses) [24], CDDO-Me suppresses gene expression and protein levels of both AR-FL and AR-V7 in the LNCaP, C4-2B, and CWR22Rv1 (22Rv1) cells. Pre-exposure to the antioxidant N-acetyl cysteine (NAC) was able to abrogate the AR suppressive effect of CDDO-Me. Most importantly, co-treatment with physiologically achievable doses of CDDO-Me could sensitize PC cells to the cytotoxic effects of a clinically approved anti-androgen drug ENZ. Our findings thus implicate the potential of CDDO-Me as an adjunct therapy in patients with CRPC tumors; especially those overexpressing AR-FL and AR-V7.

## 2. Materials and Methods 

### 2.1. Cell Culture

LNCaP (an androgen-dependent PC cell line expressing only AR-FL) and 22Rv1 (an androgen-independent PC cell line expressing both AR-FL and AR-V7) were purchased from American Type Culture Collection (ATCC; Rockville, MD, USA). The C4-2B (an androgen-independent PC cell line expressing only AR-FL) cell line was obtained from Dr. Leland Chung’s lab in Cedar Sinai Medical Center (Los Angeles, CA, USA) [45]. All the three cell lines were cultured in Rosewell Park Memorial Institute (RPMI)—1640 media supplemented with 10% fetal bovine serum (FBS) (Atlanta Biologicals; Lawrenceville, GA, USA) and 1% antibiotic–antimycotic (Thermo Scientific; Waltham, MA, USA) in a humidified incubator containing 5% CO_2_ at 37 °C. The experiments were performed in a phenol-red free RPMI media supplemented with 10% charcoal-stripped FBS (CS-FBS) from Atlanta Biologicals to simulate androgen depleted conditions.

### 2.2. Reagents

MTT [3-(4,5-dimethylthiazol-2-yl)-2,5-diphenyltetrazoliumbromide] was obtained from Sigma-Aldrich (St. Louis, MO, USA). Enzalutamide (ENZ) was purchased from ApexBio (Houston, TX, USA). Cycloheximide (CHX) was bought from Cayman chemicals (Ann Arbor, MI, USA). CDDO-Me was purchased from Selleckchem (Houston, TX, USA). The drugs were dissolved in 100% DMSO and the final DMSO concentration which was used in the experiments was less than 0.1%. N-acetyl cysteine (NAC) was obtained from Santa Cruz Biotechnology (Santa Cruz, CA, USA), dissolved in water and diluted in media immediately before use. The primary antibodies including rabbit polyclonal anti-AR (N-20) (sc-816), mouse monoclonal anti-Nrf2 (437C2a) (sc-81342), and anti-GAPDH (sc-47724) were obtained from Santa Cruz Biotechnology. The horseradish peroxidase (HRP)-conjugated goat anti-rabbit (A0545) and goat anti-mouse (A9044) secondary antibodies were bought from Sigma-Aldrich (St. Louis, MO, USA). The goat antirabbit secondary antibody tagged with Texas red (T-2767) was bought from Thermo Scientific. 

### 2.3. MTT Assay 

MTT assays were carried out to determine the cell viability post treatment with the drug(s). Briefly, ~5000 cells were cultured in 96-well plates followed by synchronization in a serum free medium overnight. The viability of the cells was determined at 72 h post exposure to drug(s) with the MTT solution (5 mg/mL for 3–4 h at 37 °C). DMSO was used to solubilize the formazan crystals and the optical density (O.D.) was measured at 540 nm with µQuant spectrophotometric plate reader (Bio-Tek; Seattle, WA, USA). 

### 2.4. Western Blot Analysis

The radioimmunoprecipitation assay (RIPA) lysis buffer (Santa Cruz Biotechnology) was used to harvest the whole cell lysates post exposure to drug(s). Quantification of the total protein was done using the bicinchoninic acid (BCA) protein assay reagent (Thermo Scientific). In brief, 10 µg of protein was electrophoresed in SDS-PAGE gels (10%) and transferred onto nitrocellulose membranes using semi-dry electro-transfer. The membranes were incubated overnight with the primary antibodies against AR (1:500 dilution), Nrf2 (1:500 dilution), and GAPDH (1:3000 dilution) at 4 °C after blocking with 5% casein in TBS-T buffer (tris buffer saline with 0.1% tween-20). The membranes were then incubated with the corresponding HRP-conjugated secondary antibodies (1:2000 dilution) for 1 h and developed using the Supersignal west femto substrate (Thermo Scientific). The scanning of the immunoblots was done using the ImageQuant LAS 500 scanner (GE Healthcare; Princeton, NJ, USA). Image J software (NIH; Bethesda, MD, USA) was used to quantify the band intensities. The densitometric values for AR proteins (AR-FL and AR-V7) were normalized to the GAPDH values for calculating the fold change.

### 2.5. ROS Assay

DCFDA/H2DCFDA—a cellular ROS assay kit (Abcam; Cambridge, MA, USA; Cat # ab113851) was used to measure reactive oxygen species (ROS). Cells were harvested and seeded in a dark, clear bottom 96-well microplate with 25,000 cells per well. The cells were stained with 2′,7′-dichlorofluorescin diacetate (DCFDA) and treated with different agents for the specified period of time. The DCFDA fluorescence (Ex/Em = 485/535 nm) was measured immediately using a microplate reader (Bio-Tek).

### 2.6. Wound-Heal Assay

Wound-heal assay was performed to monitor the migratory phenotype of PC cells post exposure to drug(s) [46]. In brief, cells were cultured in 6-well plates (1 × 10^6^ cells per well) until a confluent monolayer was formed. A 200 µl pipette tip was used to scratch the monolayer. The wells were then washed with PBS and images (10× magnification) were captured of the wound at 0 time point with a Leica Microsystems microscope (Buffalo Grove, IL, USA). Images of the wound were then captured at 72 h post exposure to the drug(s). The cell migration (wound closure) was measured by calculating the distance between 4–5 random points within the wound edges.

### 2.7. Colony Forming Units Assay

Cells (500/dish) were cultured in 60 mm petri dishes in three replicates in 2% FBS containing media and exposed to the drug(s) after 48 h. The drug(s) were replenished in the second week. After two weeks, the cell colonies were stained with 0.2% crystal violet in 20% methanol post fixation with 100% ethanol. The colony forming units (CFU) were counted with the Image J software. The total number of CFUs were then compared in untreated (control) and drug-treated cultures.

### 2.8. Immunofluorescence Microscopy

Immunofluorescence microscopy (IFM) was used to visualize subcellular localization of AR post exposure to CDDO-Me. In brief, cells (3 × 10^4^) were cultured in chamber slides (EMD Millipore; Billerica, MA, USA) and then fixed in ice cold methanol after treatment. After permeabilization of the cells with 0.1% Triton-X 100, blocking was done in 10% goat serum. The cells were then incubated with the primary antibody (1:300 dilution) overnight at 4 °C. This was followed by incubation with the corresponding secondary antibody tagged with texas red (1:1000 dilution) for 1 h. The cover slips were mounted after nuclear stain diamino-2-phenylindole (DAPI) containing vectashield mounting media (Burlingame, CA, USA) was added to the slides. The images (60× magnification) were captured with the fluorescent microscope (Leica Microsystems; Buffalo Grove, IL, USA).

### 2.9. Quantitative RT-PCR

The mRNA levels for both AR-FL and AR-V7 were measured using the quantitative reverse transcriptase polymerase chain reaction (qRT-PCR). In brief, after treatment, total mRNA was extracted using the RNeasy mini plus kit (Qiagen; Valencia, CA, USA) in accordance with the manufacturer’s instructions. The iScript cDNA synthesis kit (Bio-Rad, Hercules, CA, USA) was used to prepare complementary DNA (cDNA) according to the manufacturer’s instructions. The following primer sequences were used: (1) AR-FL:—forward: 5′-CAGCCTATTGCGAGAGAGCTG-3′ and reverse: 5′-GAAAGGATCTTGGGCACTTGC-3′; (2) AR-V7:—forward: 5′-CCATCTTGTCGTCTTCGGAAATGTTATGAAGC-3′ and reverse: 5′-TTTGAATGAGGCAAGTCAGCCTTTCT-3′; and (3) β-actin:—forward: 5′TGAGACCTTCAACACCCCAGCCATG-3′ and reverse: 5′-GTAGATGGGCACAGTGTGGGTG-3′. The iQ^TM^ SYBR green supermix (Bio-Rad) was used to measure the AR transcript levels and C1000^TM^ Thermocycler (CFX96; Bio-Rad) was used to carry out the amplification reactions. The following amplification conditions were used: Priming at 95 °C for 5 min, and then 35 cycles of 95 °C for 30 s, 55 °C for 30 s, and 72 °C for 30 s. The data (∆Ct values) for AR (AR-FL and AR-V7) transcript levels were normalized to the corresponding β-actin values for calculating the fold change.

### 2.10. Statistical Analysis

The graphPad Prism (version-6) Software (San Diego, CA, USA) was used for the statistical analyses. Results were expressed as the standard error of the mean (± SEM). A two-tailed student’s *t*-test was used to determine significant changes from controls and *p*-values of < 0.05 were considered significant. The CompuSyn software (ComboSyn, Inc., Paramus, NJ, USA) was used for synergy determination and combination index (CI) was calculated on the basis of Chou–Talalay method, which determines additive (CI = 1), synergistic (CI < 1), or antagonistic (CI > 1) effects quantitatively [47]. 

## 3. Results

### 3.1. Exposure to Low-Dose CDDO-Me Decreases AR-FL and AR-V7 Protein Levels in PC Cells, in a Time- and Dose-Dependent Manner

**Figure 1:**
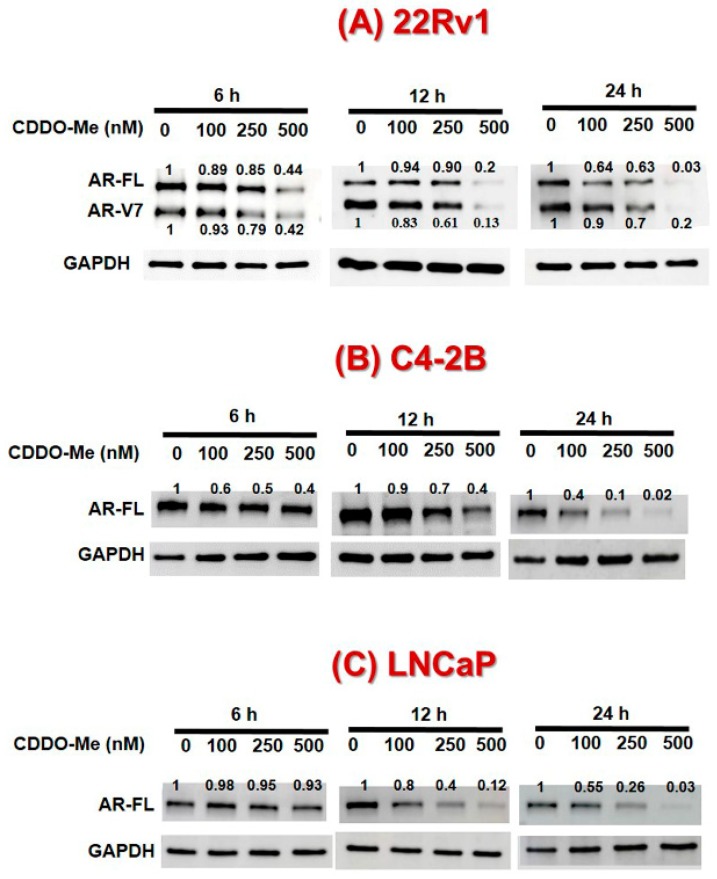

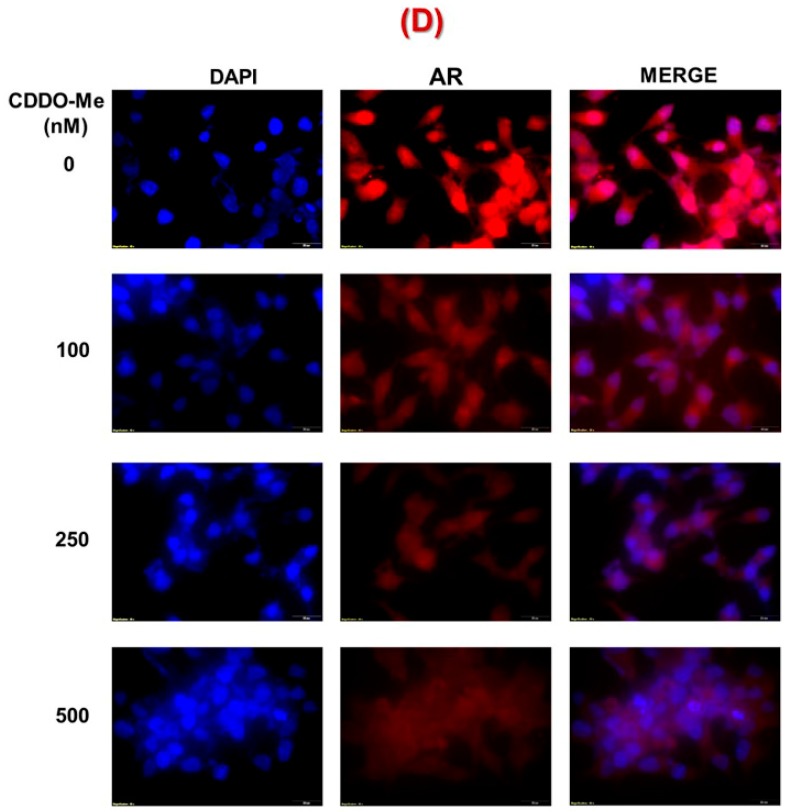
Effect of Bardoxolone-methyl (CDDO-Me) on androgen receptor (AR) levels in prostate cancer (PC) cells. 22Rv1, C4-2B, and LNCaP cells were treated with increasing concentrations of CDDO-Me (0–500 nM) and cell lysates were harvested at 6–24 h post treatment. A representative immunoblot of AR and GAPDH protein levels are shown for (**A**) 22Rv1 (**B**) C4-2B, and (**C**) LNCaP cells. (**D**) Immunofluorescence microscopy (IFM) images (60× magnification) of subcellular AR localization in PC cells. 22Rv1 cells were treated with increasing doses of CDDO-Me (100, 250, and 500 nM) for 24 h prior to fixation and immunolabeling. Left panels show DAPI stained nuclei (blue), middle panel shows AR immunoreactivity (red), and merged images are shown in the third panel.

The PC cells (LNCaP, C4-2B, and 22Rv1) were exposed to increasing concentrations of CDDO-Me (0–500 nM) and AR protein levels were measured at different time points (Figure 1A–C). Immunoblot analysis depicted that exposure to CDDO-Me causes time- and dose-dependent decreases in AR-FL in both LNCaP and C4-2B cells. Most interestingly, in 22Rv1 cells nanomolar (nM) doses of CDDO-Me were able to decrease both AR-FL and AR-V7 protein levels. In all the three cell lines, the reduction in the AR-FL and AR-V7 protein was apparent within 6 h of exposure to CDDO-Me, and was evident even with the lowest dose of CDDO-Me used (100 nM). At 24 h post exposure to the highest dose of CDDO-Me (500 nM), the AR-FL, and AR-V7 proteins were abrogated in all three cell lines. Indeed, these results were further corroborated by our IFM data, which showed clearly reduced levels of both cytoplasmic and nuclear AR immunofluorescence in the 22Rv1 cells post 24 h exposure to increasing doses of CDDO-Me (100–500 nM) (Figure 1D).

### 3.2. CDDO-Me-Mediated Suppression of AR-FL and AR-V7 is Regulated at both mRNA and Protein Levels

**Figure 2:**
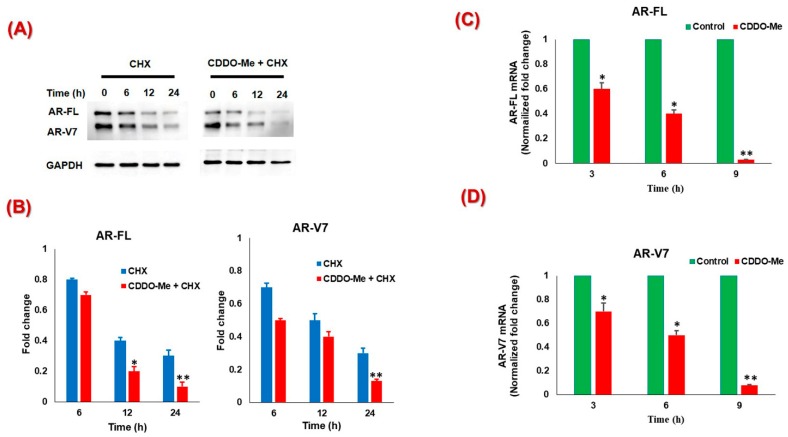
Transcriptional and post translational regulation of AR by CDDO-Me. (**A**) 22Rv1 cells were pretreated (2 h) with 5 μg/mL cycloheximide (CHX) followed by exposure to CDDO-Me (500 nM) for 0–24 h and AR levels were monitored by western immunoblot. A representative immunoblot of AR and GAPDH protein levels in 22Rv1 cells. (**B**) The normalized data are expressed as fold changes (mean ± SEM) in two independent experiments and significant differences between groups are shown as *p < 0.05. (**C,D**) 22Rv1 cells were treated with CDDO-Me (500 nM), total RNA extracted after 3, 6, and 9 h and quantitative RT-PCR (qRT-PCR) was performed. The normalized fold change in (**C**) AR-FL and (**D**) AR-V7 gene expression from two independent experiments is expressed as the mean ± SEM. Significant differences between groups are shown as *p*-values (*p < 0.05; **p < 0.005).

To determine whether the CDDO-Me-mediated suppression of AR is regulated at the level of transcription or protein synthesis, we quantified AR-FL and AR-V7 specific mRNA by qRT-PCR and AR protein levels in the presence of the protein synthesis inhibitor, cycloheximide (CHX). Immunoblot analysis revealed that combined exposure to CHX and CDDO-Me reduced the half-life of AR-FL and AR-V7 protein more significantly as compared to the CHX treatment alone(Figure 2A,B). This suggested that CDDO-Me regulates both AR-FL and AR-V7 levels at the translational level, possibly by promoting protein degradation (data not shown). Interestingly, the qRT-PCR data also showed that CDDO-Me treatment can significantly suppress the mRNA levels of both AR-FL and AR-V7 in a time dependent manner (3, 6, and 9 h) in 22Rv1 cells (Figure 2C,D). As much as 8–10-fold decrease in AR specific message was clearly observed in cells that were exposed to CDDO-Me (500 nM) for 9 h. Thus, the potent AR-suppressive effect of low-dose CDDO-Me is regulated at both transcriptional and translational levels.

### 3.3. The Suppression of AR-FL and AR-V7 by CDDO-Me is Primarily Mediated via Oxidative Stress in both C4-2B and 22Rv1 Cells

**Figure 3:**
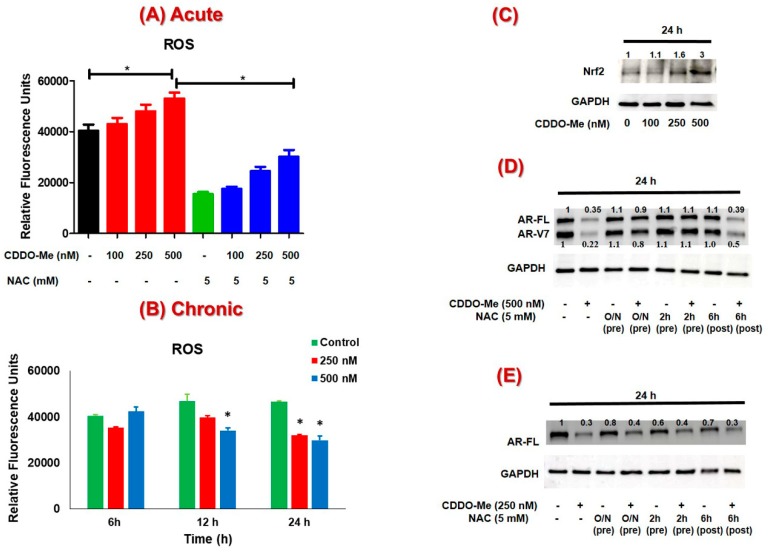
Effect of CDDO-Me mediated reactive oxygen species (ROS) on AR levels in PC cells. (**A**) Acute effect of CDDO-Me on ROS levels in 22Rv1 cells. 22Rv1 cells were exposed to CDDO-Me (100, 250, and 500 nM) for 2 h with and without 5 mM N-acetyl cysteine (NAC) (2 h pretreatment) and ROS levels were measured. (**B**) Chronic effect of CDDO-Me on ROS levels in 22Rv1 cells. 22Rv1 cells were treated with CDDO-Me (250 and 500 nM) and ROS levels were detected at 6, 12, and 24 h. The data (% of control) are expressed as the mean ± SEM of three independent experiments (*n* = 3) and significant differences between groups are shown as *p*-values (*p < 0.05) (**C**) Effect of CDDO-Me on Nrf2 protein levels. C4-2B cells were treated with increasing doses of CDDO-Me (100, 250, and 500 nM) for 24 h and total Nrf2 and GAPDH levels were detected by immunoblot. In (**D**) and (**E**), CDDO-Me exposure was carried out in cells that were either pretreated (2 h or overnight (O/N)) or posttreated (6 h) with NAC. Cell lysates were obtained at 24 h post CDDO-Me treatment of (**D**) 22Rv1 or (**E**) C4-2B cells. A representative immunoblot of AR and GAPDH protein levels is shown.

Several studies have shown that oxidative stress signaling can regulate AR expression and CRPC progression [48,49]. Antioxidant agents have also been reported to activate the Nrf2 transcription factor by transient induction of ROS [50,51]. Therefore, we wanted to determine if CDDO-Me, which is a potent antioxidant agent and a well-known inducer of Nrf2 [24], can similarly induce oxidative stress and Nrf2 in PC cells. Exposure to CDDO-Me exerted a biphasic effect on ROS levels in the 22Rv1 cells. Acute exposure to CDDO-Me (2 h) was found to increase ROS in a dose-dependent manner, which could be blocked by co-exposure of cells with the antioxidant agent, N-acetyl cysteine (NAC) (Figure 3A). Interestingly, however at 6, 12, and 24 h post exposure to CDDO-Me, even the basal ROS levels were found to decrease considerably (Figure 3B), possibly due to the activation of the Nrf2 pathway. This hypothesis was corroborated by an increase in the total levels of Nrf2 protein in the C4-2B cells, where the dose-dependent increase in Nrf2 was evident post 24 h exposure to CDDO-Me (Figure 3C).

To determine whether transient induction of ROS was important for the AR-suppressive effect of CDDO-Me, the 22Rv1 cells were exposed to NAC both pre and post treatment with CDDO-Me for 24 h (Figure 3D). Pretreatment with NAC (5 mM) for overnight or even 2 h before CDDO-Me addition was able to abrogate the AR-suppressive effects of CDDO-Me (500 nM). Interestingly however, exposure to NAC at 6 h post treatment with CDDO-Me was not able to abolish its AR suppressive effects at 24 h. These findings suggested that the acute induction of ROS, observed within 2 h post exposure to CDDO-Me, was critical in decreasing the levels of AR-FL and AR-V7 in 22Rv1 cells. Similar results could be seen in C4-2B cells as well, where only NAC pretreatment, but not post treatment, was able to nullify the AR-suppression by CDDO-Me (Figure 3E).

### 3.4. Co-Exposure to CDDO-Me Increases the Anticancer Efficacy of ENZ 

**Figure 4:**
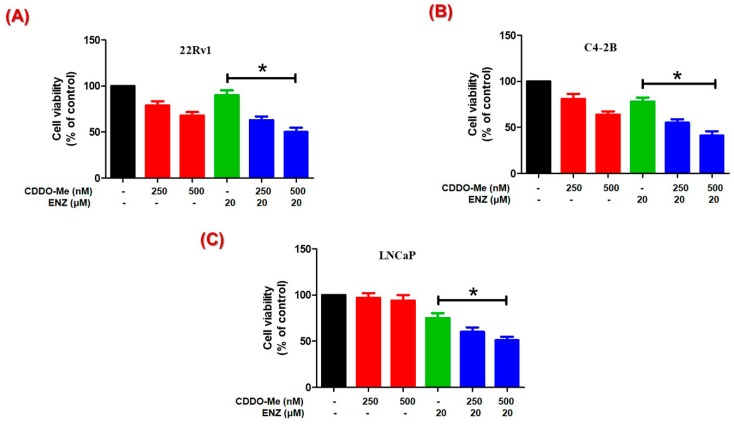
Effect of CDDO-Me and enzalutamide (ENZ) combination on cell viability of PC cells. Cytotoxic effect of CDDO-Me (250 and 500 nM) alone and in combination with ENZ (20 μM) at 72 h post treatment in (**A**) 22Rv1, (**B**) C4-2B, and (**C**) LNCaP cells is shown. PC cells were treated with CDDO-Me alone and in combination with ENZ and cell viability was measured at 72 h using MTT. The data (% of control) are expressed as the mean ± SEM of three independent experiments (*n* = 3) and significant differences between groups are shown as *p*-values (*p < 0.05).

To determine whether the AR-suppression by CDDO-Me enhances the efficacy of clinically approved anti-androgens, cytotoxicity was measured in LNCaP, C4-2B, and 22Rv1 cells co-exposed to CDDO-Me and ENZ, using the MTT cell viability assay(Figure 4). Cell viability data at 72 h clearly showed that the 22Rv1 cells, which expresses both AR-FL and AR-V7, were more sensitive to CDDO-Me than the C4-2B cells, which expresses AR-FL only (Figure 4A,B). The LNCaP cells, which express AR-FL and are androgen responsive, were least sensitive to CDDO-Me even at the highest dose tested (500 nM) (Figure 4C). Most importantly, we observed that co-exposure to CDDO-Me was able to increase the therapeutic efficacy of ENZ in the 22Rv1 cells. These CRPC cells are resistant to anti-androgens, and ENZ treatment alone showed negligible decreases in cell viability. However, CDDO-Me alone was shown to decrease cell growth and co-exposure to CDDO-Me was able to further augment the anticancer efficacy of ENZ in 22Rv1 cells. Similar enhancements in cytotoxicity following CDDO-Me and ENZ co-exposure were also evident in the LNCaP and C4-2B cells.

### 3.5. Co-Exposure to CDDO-Me and ENZ Abrogates the Migratory Potential of CRPC Cells

**Figure 5:**
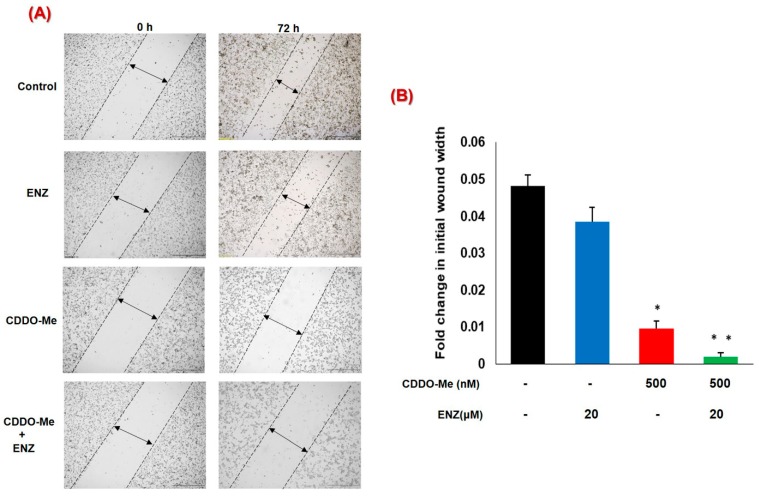
Effect of combination of CDDO-Me and ENZ on cell migration in 22Rv1 cells. Quantification of cell migration was examined by wound-healing assay. (**A**) A representative light microscope image of the wound at the 0 and 72 h time-points is shown in 22Rv1 cells. Images show wound closure in untreated (control) cells, and in cultures that were exposed to either CDDO-Me (500 nM), ENZ (20 μM), or CDDO-Me and ENZ combination. (**B**) Fold change in wound width is expressed as the mean ± SEM of two independent experiments, and significant differences between groups are shown as *p*-values (* *p* < 0.05; **p < 0.005).

Increased metastatic ability of CRPC cells has been linked to increased AR signaling [52] and can be tested in vitro by using different migration and invasion assays [46]. We carried out wound-heal assays to determine the effect of CDDO-Me, alone, or in combination with ENZ, on the migratory behavior of 22Rv1 cells (Figure 5A,B). The vigorous migratory ability of 22Rv1 cells was clearly evident by as much as a 50% reduction in wound-width within 72 h post wounding. Exposure to ENZ alone failed to show significant inhibition in migration of these aggressive CRPC cells. However, exposure to CDDO-Me alone significantly reduced cell migration and the combined treatment with CDDO-Me and ENZ almost totally abrogated the migratory potential of 22Rv1 cells.

### 3.6. CDDO-Me Increases ENZ Efficacy by Inhibiting the Clonogenic Ability of PC Cells

**Figure 6:**
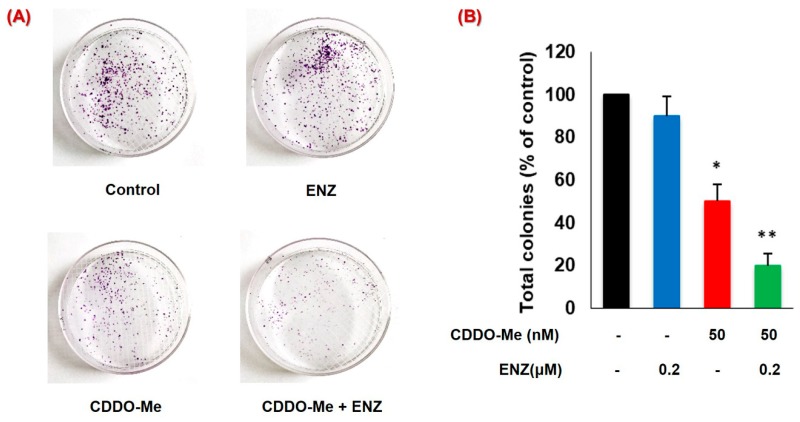
Long-term effects of CDDO-Me and ENZ on the clonogenic ability of PC cells. The 22Rv1 cells (500 cells/plate) were exposed to CDDO-Me (50 nM) alone or in combination with ENZ (0.2 μM) for two weeks. (**A**) A representative image of colony forming units (CFU) is shown. (**B**) Effect of drug exposure on percent change in total number of CFUs in 22Rv1 cells as compared to the control is shown. Data are expressed as the mean ± SEM of two independent experiments and significant differences between groups are shown as *p*-values (*p < 0.05; **p < 0.005).

The clonogenic ability of CRPC cells is a major determinant of metastatic growth [53]. We carried out the colony forming unit (CFU) assay to investigate the chronic effects (14 days) of low-dose CDDO-Me, alone and in combination with ENZ, on the clonogenic ability of 22Rv1 cells (Figure 6A,B). Interestingly, treatment with ENZ alone (0.2 μM) did not cause any significant inhibition in the total number of CFUs. However, chronic exposure to even low-dose CDDO-Me (50 nM) caused approximately 50% reduction in the number of CFUs. Most importantly, co-exposure to CDDO-Me and ENZ enabled almost a total abrogation (~80%–90%) in the number of CFUs. The CFU suppressive effect of CDDO-Me was synergistically increased (CI < 1) when combined with low-dose ENZ, which alone did not significantly alter the CFUs. A remarkable suppression in CFUs generated by the 22Rv1 cells was evident following chronic exposure to very low-doses CDDO-Me and ENZ thus underscoring the therapeutic potential of this novel combination. 

## 4. Discussion

Second messenger signaling via the androgen receptor is indispensable for the prostate epithelial cells, not only for the normal functioning and homeostasis of the prostate gland but also for the development of prostatic neoplasms and the progression of PC to CRPC [2]. Therefore, therapeutic strategies to suppress AR signaling have remained the mainstay of PC treatment [5]. Despite the initial efficacy of ADT, PC patients eventually become resistant to even high doses of antiandrogens, leading to the development of CRPC phenotype [2]. One of the most crucial factors implicated in the emergence of CRPC cells is the expression of constitutively active AR splice variants (AR-Vs); AR-V7 being predominantly documented in clinical samples [12]. Interestingly, AR-FL is still persistently expressed in the CRPC cells, and is known to dimerize with truncated AR splice variants [22,23]. Furthermore, the crosstalk of AR with other signaling pathways involved in tumorigenesis results in the synergistic aberrant expression of the target genes associated with mitogenesis, increased cell viability, clonogenicity, and migratory behavior, leading to aggressive and invasive CRPC recurrence [54,55]. Therefore, therapeutic agents which can suppress both AR-FL and AR-V7 at physiologically achievable concentrations are urgently needed. Recently, we had documented that sulforaphane (SFN), a phytochemical derived from broccoli sprouts, and other cruciferous vegetables, can suppress the expression of both AR-FL and AR-V7 [56,57]. This AR-suppressive effect of SFN, a known Nrf2 inducer, was also shown to enhance the anticancer efficacy of anti-androgens [56,57]. Since CDDO-Me is also a potent Nrf2 inducer, in this study, we investigated the effect of CDDO-Me on AR expression in CRPC cells. Our findings demonstrated that low-dose CDDO-Me suppresses both AR-FL and AR-V7 expression and augments the efficacy of the second-generation anti-androgen, ENZ. At nanomolar concentrations (100–500 nM), exposure to CDDO-Me suppressed the protein expression of AR-FL in the androgen dependent LNCaP cells and in the androgen independent C4-2B cells [Figure 1]. In addition, it also inhibited the protein expression of both AR-FL and AR-V7 in the highly aggressive CRPC cell line, 22Rv1 [Figure 1]. Since CDDO-Me is in late-stage clinical trials for chronic kidney disease (CKD), we envision that its utility as an adjunct to ADT will be very valuable in treating CRPC and should be clinically tested. 

LNCaP is an androgen-dependent cell line that was first isolated from a human metastatic prostate adenocarcinoma in the lymph node and expresses mRNA/protein of both AR and PSA [58,59]. C4-2B is a bone metastatic CRPC subline derived from LNCaP which also expresses both AR and PSA although it also can grow in androgen deprived conditions [45,59]. C4-2B cells have been shown to grow both in intact, as well as castrated mice. 22Rv1 is a CRPC cell line expressing AR-FL and several AR splice variants, out of which AR-V7 is the most prominent [59]. It was isolated from the xenograft CWR22R derived from a patient with bone metastasis. In vitro studies using these three PC lines provide a model for both the early stages of PC and the progression of CRPC cells to AR-variant expressing aggressive and hormone-resistant phenotype. Thus, our observations on the potent suppression of both AR-FL and AR-V7 by the low-dose CDDO-Me implicate its potential to be used in both the early stage PC where the cancer cells are hormone dependent, as well as in the late stage of CRPC tumors where the cancer cells have selected for an androgen independent phenotype, but are still expressing AR-FL and AR-V7. 

The CDDO-Me-mediated suppression of AR-FL and AR-V7 was found to be regulated both at the transcriptional, as well as the translational level [Figure 2]. Indeed, suppression of AR-FL and AR-V7 protein levels by combination of CDDO-Me and CHX was more significant as compared to the CHX treatment alone, suggesting that post translational regulation of AR may be a possible mechanism of action of CDDO-Me in these cells. We documented in our previously published study, that SFN can increase proteasomal degradation of AR [57]. Likewise, our unpublished observations suggest increased proteasomal activity in CDDO-Me exposed cells. CDDO-Me on binding to sulfhydryl (SH) groups of cysteine residues [60,61] on DNA binding domain (DBD) of AR-FL and AR-V7, may also increase the accumulation of misfolded AR proteins [62]. Moreover, since CDDO-Me has been shown to directly interact with HSP90 and degrade HSP90 client proteins [63], this may also be one of the mechanisms responsible for the AR suppressive effect of CDDO-Me in PC cells since AR is one of the client proteins of HSP90 [64]. Most interestingly, a significant decrease in mRNA levels of both AR-FL and AR-V7 was evident even at 3 h post exposure to CDDO-Me, suggesting that second messenger signaling by CDDO-Me may alter the transcriptional machinery at the AR promoter/enhancer regions, and thus rapidly downregulate AR gene expression. We had previously documented that overexpression of Nrf2 can suppress AR expression and function in LNCaP and C4-2B cells [65]. Exposure to the Nrf2 inducer, SFN, can also decrease AR gene expression [56]. Thus, transcriptional suppression of AR gene expression via Nrf2 inducers may be a novel direction in PC therapy. Ultimately, our observations on the dual effects of CDDO-Me on AR suppression may be highly beneficial in the long-term ablation of AR mediated protumorigenic effects in aggressive PC cells.

**Figure 7:**
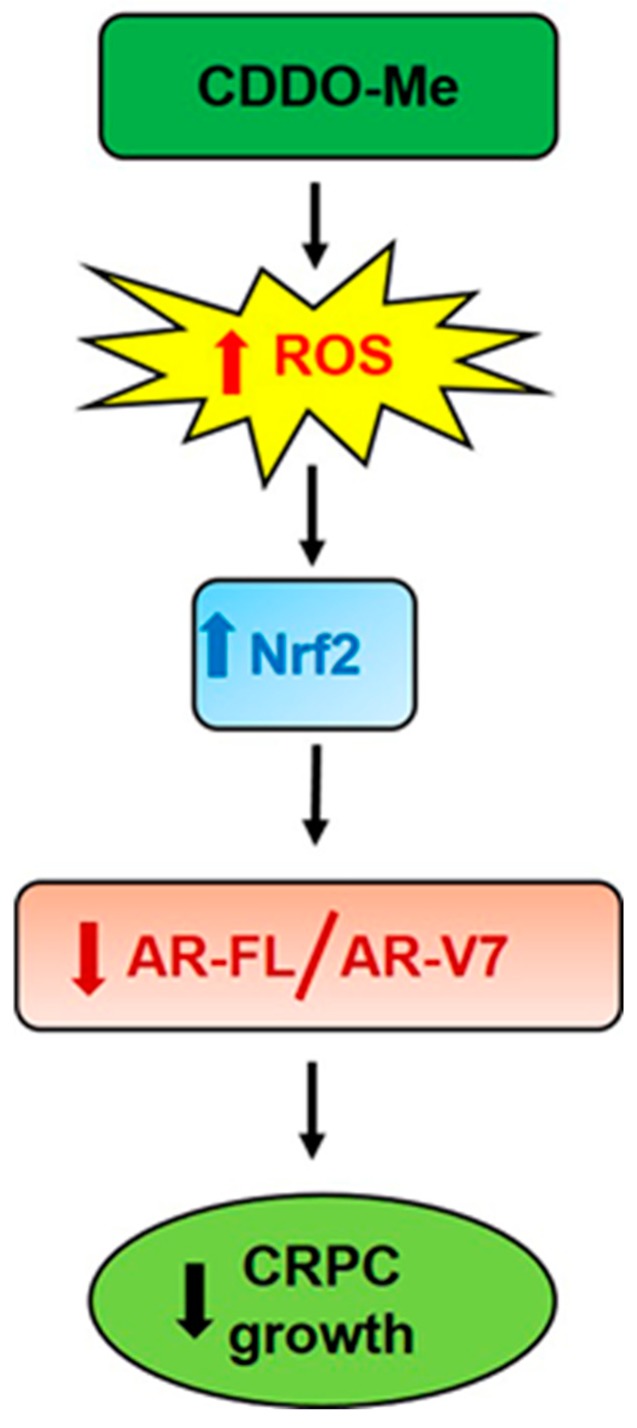
Mechanism of AR suppressive actions of CDDO-Me in PC cells. Exposure to Bardoxolone-methyl (CDDO-Me) causes a transient induction of reactive oxygen species (ROS) which in turn activates Nrf2 protein levels. Ultimately, this antioxidant transcription factor decreases oxidative stress and the aggressive properties of CRPC cells, e.g., growth, migration, clonogenic ability, etc. Both Nrf2 and ROS may be directly involved in suppressing AR expression (both at the gene and protein levels). Decreased AR signaling (via AR-FL and AR-V7) by CDDO-Me inhibits the growth of CRPC cells and can augment the anticancer efficacy of approved drugs like enzalutamide (ENZ).

Several other Nrf2 inducing antioxidant phytochemicals such as SFN and curcumin have promising anticancer effects and are implicated to function by first inducing oxidative stress followed by increasing nuclear Nrf2 levels [66,67,68,69]. Similarly, CDDO-Me, being a much more potent Nrf2 inducing agent, has also been shown to rapidly induce ROS to cause cytotoxicity in various cancer cell lines in vitro [51]. In our studies, we similarly observed a biphasic effect of CDDO-Me on ROS production in PC cells [Figure 3A,B]. Exposure to CDDO-Me caused a transient induction of ROS within 2 h, whereas it suppressed ROS levels in the long-term (24 h), most likely by increasing Nrf2 levels [Figure 3C]. Nrf2 protein levels were found to be significantly increased at 24 h post exposure to CDDO-Me. Most importantly, the transient induction of ROS was found to be crucial for the potent AR suppressive effect of CDDO-Me on both AR-FL and AR-V7 expression. This was clearly evident from the rescue experiments, where pretreatment with NAC was able to nullify the AR suppressive effects of CDDO-Me [Figure 3D,E]. Post treatment with NAC, even within 6 h following CDDO-Me exposure, was not able to salvage the suppression of AR. Therefore, in addition to the role of Nrf2, a direct effect of oxidative stress in regulating both AR-FL and AR-V7 mRNA and protein in CRPC cells is possible. Oxidative stress has been associated with changes in the expression of several splicing factors, e.g., the hetero-nuclear ribonucleoproteins (hnRNPs) [70]. HnRNPs are important for promoting AR expression and production of variants in PC [71]. A putative molecular mechanism/s linked to the therapeutic effects of CDDO-Me is presented in Figure 7. 

Importantly, functional assays addressing the tumorigenic ability of PC cells, i.e., viability, migration and clonogenicity, clearly demonstrated that the AR-suppressive effects of CDDO-Me can enhance the efficacy of the clinically approved anti-androgen ENZ. Exposure to nanomolar doses of CDDO-Me was able to potentiate the efficacy of ENZ in LNCaP, C4-2B, and the ENZ resistant 22Rv1 cells by significantly decreasing the cell viability of PC cells [Figure 4]. Androgen signaling enhances the migratory behavior of PC cells [2]. Higher invasive ability of cells, augmented apoptotic resistance, and epithelial mesenchymal transition (EMT) are the chief characteristics of migration, which are also critical elements of tumor metastasis [46,72]. In this respect, although antiandrogens have potent antiproliferative effects, they do not suppress PC cell migration significantly [73]. Our study shows that CDDO-Me treatment can decrease the migratory ability of PC cells and this effect is further augmented in the presence of ENZ [Figure 5]. Our in vitro finding thus advocates the benefit of using CDDO-Me in combination with ENZ to inhibit the metastatic behavior of PC cells. These findings may be of significant therapeutic value, especially in CRPC patients that overexpress AR-FL and/or AR-V7 and continue to utilize the AR signaling pathway. 

Although we have not carried out in vivo studies in tumor-bearing animals, the potent antitumor effects seen with our drug combination were validated by measuring the clonogenic ability of PC cells in vitro [Figure 6]. Exposure to even a very low-dose of CDDO-Me (50 nM) showed significant suppression of CFUs, and co-exposure to CDDO-Me significantly enhanced the ability of ENZ to suppress the number of colonies. In vitro clonogenic assays simulate the seeding and proliferation of tumor initiating cells, i.e., cancer stem cells [74]. A number of previous studies have already been carried out in tumor xenografts in vivo using either clinically approved ENZ [75,76,77,78,79] or CDDO-Me [26,37,40,41,44] alone; and therefore, studies using the combination of CDDO-Me and ENZ in tumor-bearing mice, would be the next feasible direction of our current findings. Taken together, our in vitro finding suggests a possible benefit of using safe concentrations of CDDO-Me alone to suppress the growth of micro-metastatic foci, and as an adjunct therapy to enhance the efficacy of antiandrogens. The long-term synergistic effects observed in our CFU assays, especially with very low doses of each of the agents, suggest the potential of this combination therapy. 

## 5. Conlusions

Nanomolar (nM) concentrations of Bardoxolone-methyl (CDDO-Me) decreased both AR-FL and AR-V7 expression in PC cells. Therefore, CDDO-Me, which is in several late-stage clinical trials, may be used in combination with clinically approved anti-androgens such as ENZ in PC patients; both at the initial stage where tumors are overexpressing AR-FL only and at the later stage of CRPC where AR-V7 overexpressing tumors are frequently observed.
